# Treatment with robot-assisted gait trainer Walkbot along with physiotherapy vs. isolated physiotherapy in children and adolescents with cerebral palsy. Experimental study

**DOI:** 10.1186/s12883-024-03750-9

**Published:** 2024-07-15

**Authors:** Raquel Olmos-Gómez, Inmaculada Calvo-Muñoz, Antonia Gómez-Conesa

**Affiliations:** 1 International Doctoral School of the University of Murcia (EIDUM), University of Murcia, Murcia, 30100 Spain; 2 Faculty of Physiotherapy, Occupational Therapy and Podiatry, UCAM Catholic University of Murcia, Guadalupe, Murcia, 30107 Spain; 3 Research Group Research Methods and Evaluation in Social Sciences, Mare Nostrum Campus of International Excellence, University of Murcia, Murcia, 30100 Spain

**Keywords:** Cerebral palsy, Robotic, Walkbot, Gait, Children, Adolescents, Experimental study, Physical therapy modalities

## Abstract

**Background:**

Improving walking ability is a key objective in the treatment of children and adolescents with cerebral palsy, since it directly affects their activity and participation. In recent years, robotic technology has been implemented in gait treatment, which allows training of longer duration and repetition of the movement. To know the effectiveness of a treatment with the robotic-assisted gait trainer Walkbot combined with physiotherapy compared to the isolated physiotherapy treatment in children and adolescents with cerebral palsy, we carried out a clinical trial.

**Methods:**

23 participants, were divided into two groups: experimental and control. During 5 weeks, both groups received their physiotherapy sessions scheduled, in addition experimental group received 4 sessions per week of 40 min of robot. An evaluation of the participants was carried out before the intervention, at the end of the intervention, and at follow-up (two months after the end of the intervention). Gait was assessed with the Gross Motor Function Measure-88 dimensions D and E, strength was measured with a hydraulic dynamometer, and range of motion was assessed using the goniometer. A mixed ANOVA was performed when the assumptions of normality and homoscedasticity were met, and a robust mixed ANOVA was performed when these assumptions were not met. Statistical significance was stipulated at *p* < 0.05. For the effect size, η^2^ was calculated.

**Results:**

Significant differences were found regarding the time x group interaction in the Gross Motor Function Measure-88 in dimension D [η^2^ = 0.016], in the flexion strength of the left [η^2^ = 0.128] and right [η^2^ = 0.142] hips, in the extension strength of the right hip [η^2^ = 0.035], in the abduction strength of the left hip [η^2^ = 0.179] and right [η^2^ = 0.196], in the flexion strength of the left knee [η^2^ = 0.222] and right [η^2^ = 0.147], and in the range of motion of left [η^2^ = 0.071] and right [η^2^ = 0.053] knee flexion.

**Conclusions:**

Compared to treatments without walking robot, physiotherapy treatment including Walkbot improves standing, muscle strength, and knee range of motion in children and adolescents with cerebral palsy.

**Trial registration:**

ClinicalTrials.gov: NCT04329793. First posted: April 1, 2020.

## Background

Cerebral Palsy (CP) is one of the main causes of locomotor and postural disorders in children, being the most common cause of physical disability in childhood [1]. Disturbances in movement and posture often lead to spasticity, muscle weakness, and disturbances in selective motor control, making walking difficult [2, 3]. Abnormal gait patterns can cause secondary deformities and reduce their quality of life, limiting the opportunity to explore the environment and restricting their participation and independence [3]. From physiotherapy there are different approaches to address this limitation in participation and activity, including the robotic-assisted gait training (RAGT) [4–6]. The RAGT provides conditions that support motor learning principles such as intensity, repetition, task specificity, and participation [7–9]; as well as presenting beneficial effects for improving walking in subjects with brain and spinal cord damage [10–14]. Some studies have investigated the effects of the RAGT in improving gait function in CP subjects, showing an overall improvement in gait parameters (mainly gait speed, stride length and frequency), endurance, and gross motor function (Gross Motor Function Measure-88 (GMFM-88) dimensions D and E [15–21] A recent network meta-analysis of clinical trials concluded that although there is evidence to suggest that RAGT treatments are effective in children and adolescents with CP, no significant difference was found between RAGT and physiotherapy treatments in improving gait and standing [22]. Among the RAGT systems, there is the Walkbot (P&S Mechanics, Seoul, Korea) which consists of an exoskeletal RAGT system that is attached to the patient and helps him move his lower extremities on a treadmill, being attached by a harness to a crane, generating a personalized gait pattern for the patient [23], and provides ambulation that is closest to human kinematics and kinetics [24]. To our knowledge, only one study has been conducted in the paediatric population with CP, investigating the effects of the Walkbot-K system and comparing two groups in which both used the robot, with significant treatment effects in dimensions D and E of the GMFM [25].

The objective of this study is to know the effectiveness of RAGT treatment with Walkbot combined with physiotherapy compared to the isolated physiotherapy treatment in children and adolescents with CP, to improve gait function, and to increase muscle strength and range of motion in the lower limbs.

## Methods

A quasi-experimental, prospective, longitudinal study was carried out. It was registered with number: NCT04329793, respecting the ethical principles of the 2013 Helsinki declaration and the ethical protocol set by the Ethics and Research Commission of the University of Murcia, being approved by them. The parents of the patients or their legal guardians were duly informed and they were given an informed consent form, which was read, understood and signed in order to participate in the study.

### Study population and recruitment

The sample was made up of children and adolescents diagnosed with CP from different reference hospitals, associations and educational guidance teams from six provinces in the south and east of Spain. The inclusion criteria were as follows: bilateral CP; age from 3 to 18 years; Gross Motor Function Classification System – Expanded & Revised (GMFCS-ER) levels II, III and IV; not having received or being receiving treatment with RAGT or partially weight-bearing walking treadmill in the last year, and acceptance of participation in the study with the signing of the informed consent. The exclusion criteria were: serious psychiatric problems that prevent placement in the robot; serious heart problems that prevent physical exercise; active tumour processes; serious degenerative joint problems; degenerative diseases of the nervous system; mitochondrial diseases; recent surgeries; ununited fractures; severe osteoporosis; uncontrolled epileptic seizures; open wounds on the lower half of the body; extreme fear of being placed in robotic devices; pain that prevents you from carrying out the treatment; and that their anthropomorphic measurements are below the minimum required to be able to use the device.

Children were assigned to group experimental (EG) or control (CG) with non-random method.

### Intervention

EG participants underwent the RAGT treatment along with their usual physiotherapy treatment, while CG participants received physiotherapy treatment without the RAGT. The EG participants received 4 treatment sessions per week consisting of 40 min in the RAGT in addition to their physiotherapy sessions that were scheduled for the same number of weeks. The total number of sessions was 20, in uninterrupted weeks. The CG participants received the scheduled physiotherapy sessions during the same weeks, performing between 3 and 5 weekly sessions. The physiotherapy treatment in both groups was similar and applied by the physiotherapists in their respective educational, health centres or associations.

### Assessments

The evaluation was carried out before starting the treatment (pretest), at the end of the treatment (posttest) and two months after finishing the intervention (follow-up). A trained physiotherapist, with 15 years of experience, and not blinded to the study conditions, carried out all the evaluations at a private clinic in Murcia or Granada (Spain), in the same conditions.

Gait was evaluated with the GMFM-88 [26]. The strength of the large muscle groups in the lower extremities in the movements of hip flexion and extension, knee flexion and extension and hip abduction were measured with the Baseline^®^ hydraulic push-pull dynamometer. The range of motion (ROM) of the joints of the lower limbs, specifically, knee flexion-extension, were evaluated by measurements carried out with the Enraf / Nonius Universal goniometer.

### Data analysis

The analyses were carried out with the free statistical package R, version 4.0.3 (R Core Team 2020). A descriptive analysis of the variables was carried out, using the mean as a measure of central tendency and the standard deviation, maximum value and minimum value as a measure of dispersion. Before performing the inferential statistics, the assumptions of normality were checked using the Kolmogorov-Smirnov test; and homogeneity using the Breusch-Pagan and Fligner-Killeen tests for homogeneity of variances, taking into account that both tests assume normality and homogeneity of variances as the null hypothesis. Durbin-Watson test was used for autocorrelation. A mixed ANOVA was performed when the assumptions of normality and homoscedasticity were met, and a robust mixed ANOVA was performed when these assumptions were not met. Statistical significance was stipulated at *p* < 0.05. For the effect size, η^2^ was calculated, with a value > 0.14 considered high; moderate between 0.14 and 0.06; and small ones between 0.06 and 0.01. To test the differences between the mean values in the initial evaluation and the final evaluation, the repeated measures ANOVA of a mixed factor was used, with an inter-subject factor and within-subjects repeated measures ANOVA.

The data analysts were always blinded and were unaware of which group each patient belonged to.

## Results

**Fig. 1:**
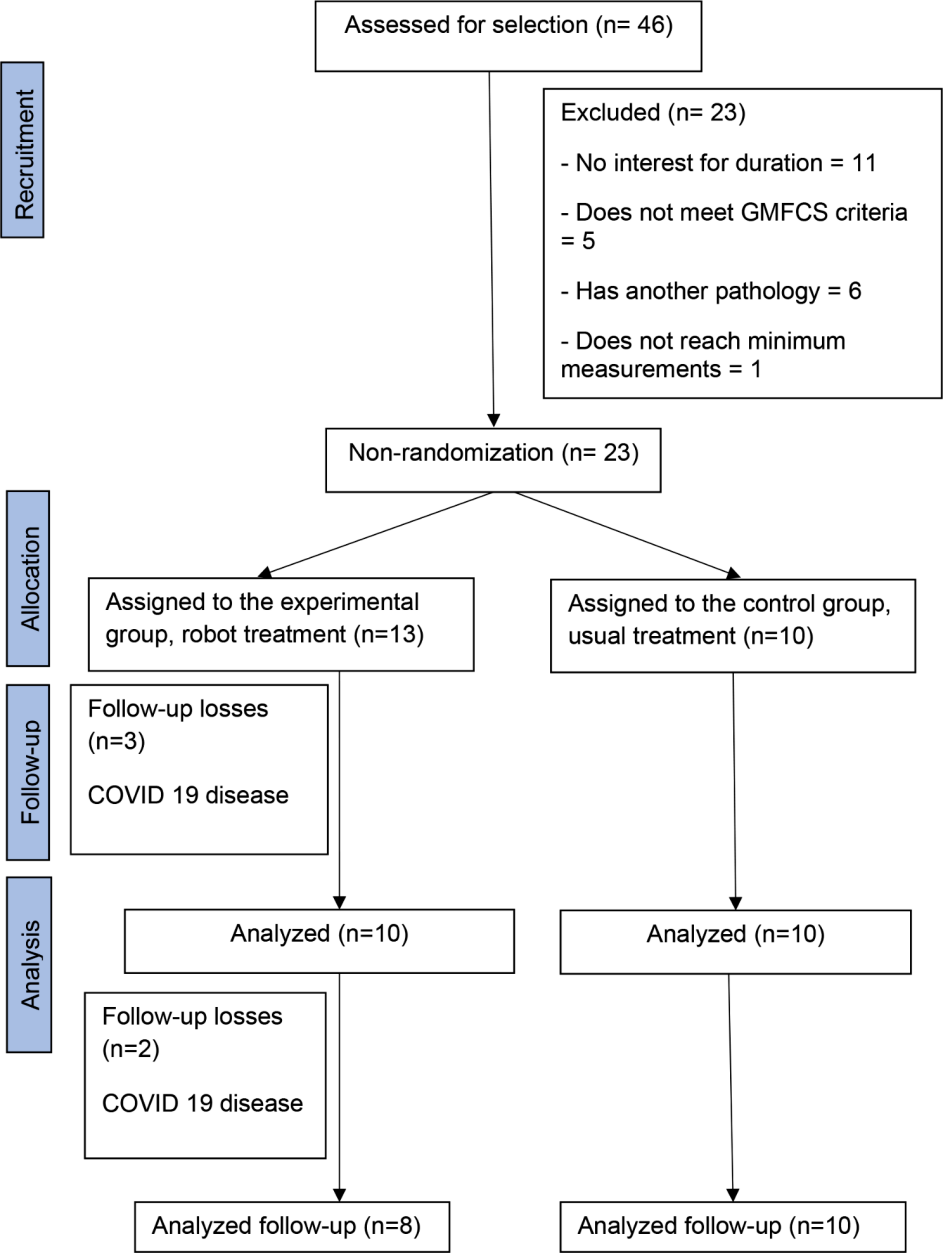
Flowchart of sample selection process

23 subjects were recruited, 13 being assigned to the experimental group (EG) and 10 to the control group (CG). Of the 13 assigned to the EG, 10 underwent the treatment and were administered pretest, posttest, and 8 follow-up measures, while the remaining 3 were evaluated in the pretest. Figure 1 shows the progress of subjects through the phases of the clinical trial. The groups were not randomized. The criterion for assignment to the EG was being able to attend the treatment sessions at the clinic where the therapy with the RAGT was performed.

Attending the classification of Surveillance of cerebral palsy in Europe [1], the 76.92% of the sample in the EG and the 90% in the CG was bilateral spastic; the rest of the sample was bilateral mixed. The characteristics of the sample are detailed in Table 1. There are no differences between groups that influence the results.

**Table 1:** Characteristics of the sample

	EG	CG
N	13	10
Mean age (sd)	7.31 (3.09)	10.20 (4.71)
Men	69.23%	90%
CP Type according to anatomical distribution		
Diplegia	46.15%	40%
Triplegia	0%	10%
Tetraplegia	53.85%	50%
Surveillance of Cerebral Palsy in Europe (SCPE) Classification [1]		
Bilateral Spastic	76.92%	90%
Mixed	23.08%	10%
GMFCS Level		
II	15.38%	50%
III	46.15%	20%
IV	38.46%	30%
Cognitive impairment		
Yes	61.54%	50%
No	38.46%	50%
Average number of weekly physiotherapy sessions (sd)	2.92 (0.95)	3.50 (1.43)

EG: experimental group; CG: control group; sd: standard deviation; CP: cerebral palsy; GMFCS: Gross Motor Function Classification System

### Gait

**Fig. 2:**
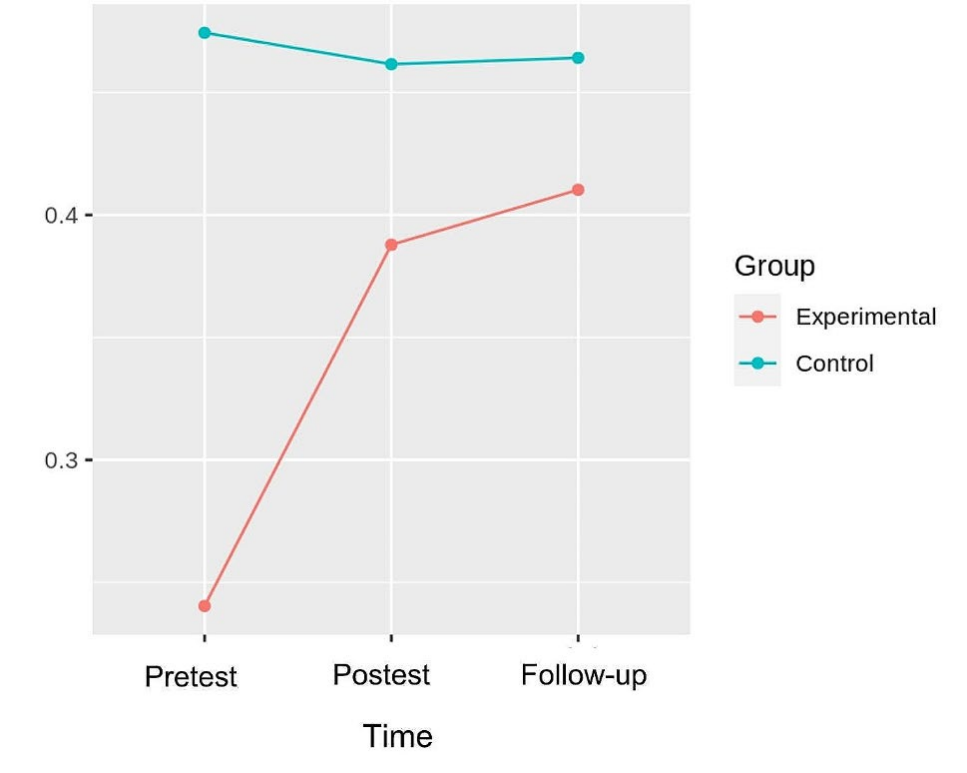
Interaction graph between time and group of GMFMD. GMFMD: Gross Motor Function Measure Dimension D

In the GMFM-88, dimension D, the pretest-post-test and follow-up analysis of variance showed differences with respect to the time x group interaction [F (2.32) = 12.758, *p* = 8.425e-05, η^2^ = 0.016], with small effect sizes. In the GMFM − 88, dimension E, in the robust mixed ANOVA pretest-posttest and follow-up, no differences were found regarding interaction (*p* = 0.158). Table 2 shows the comparisons between groups of GMFM D, GMFM E. Figure 2 shows the interaction between time and group.

**Table 2:** Comparisons between groups of GMFM D and GMFM E

	Pretest Mean (SD)		Postest Mean (SD)		Follow-up Mean (SD)		Pretest-post-test and follow-up analysis of variance	
	EG (*n* = 10)	CG (*n* = 10)	EG (*n* = 10)	CG (*n* = 10)	EG (*n* = 8)	CG (*n* = 10)	*p*	η^2^
GMFMD	0.21 (0.24)	0.47 (0.37)	0.36 (0.25)	0.46 (0.37)	0.41 (0.30)	0.46 (0.36)	8.425e-05	0.016
GMFME	0.16 (0.16)	0.44 (0.37)	0.24 (0.20)	0.47 (0.36)	0.27 (0.23)	0.44 (0.37)	0.158	--

SD: standard deviation; EG: experimental group; CG: control group; GMFMD: Gross Motor Function Measure Dimension D; GMFME: Gross Motor Function Measure Dimension E; *p*: statistical significant; η^2^: effect size

### Muscular strength

#### Hip flexion

**Fig. 3:**
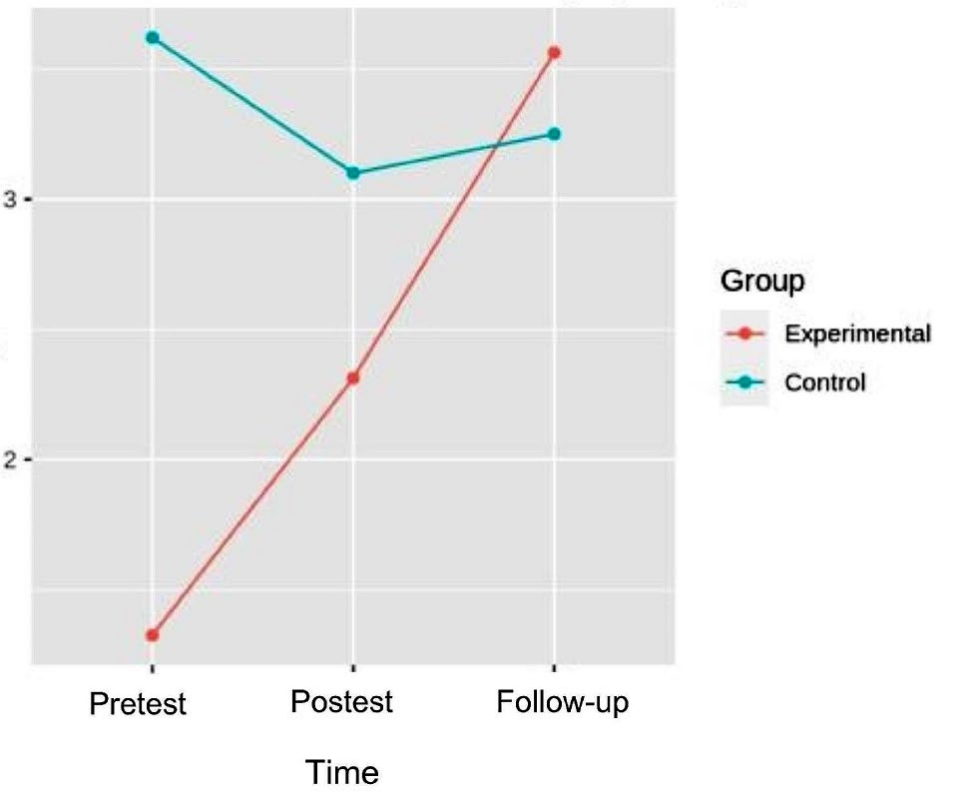
Interaction graph between time and group of left hip flexion strength

**Fig. 4:**
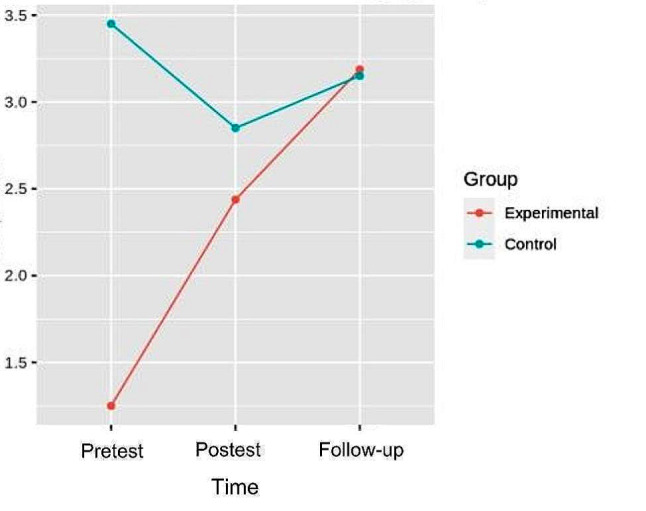
Interaction graph between time and group of right hip flexion strength

In the flexion strength, the pretest-posttest and follow-up analysis of variance showed differences with respect to the group x time interaction [F (2.32) = 7.358, *p* = 2.350e-03, η^2^ = 0.128], in left hip flexion strength. In the right hip, differences were also found with respect to the interaction [F (2.32) = 8.045, *p* = 1.478e- 03, η^2^ = 0.142]. (Figures 3 and 4). (Table 3).

#### Hip extension

**Fig. 5:**
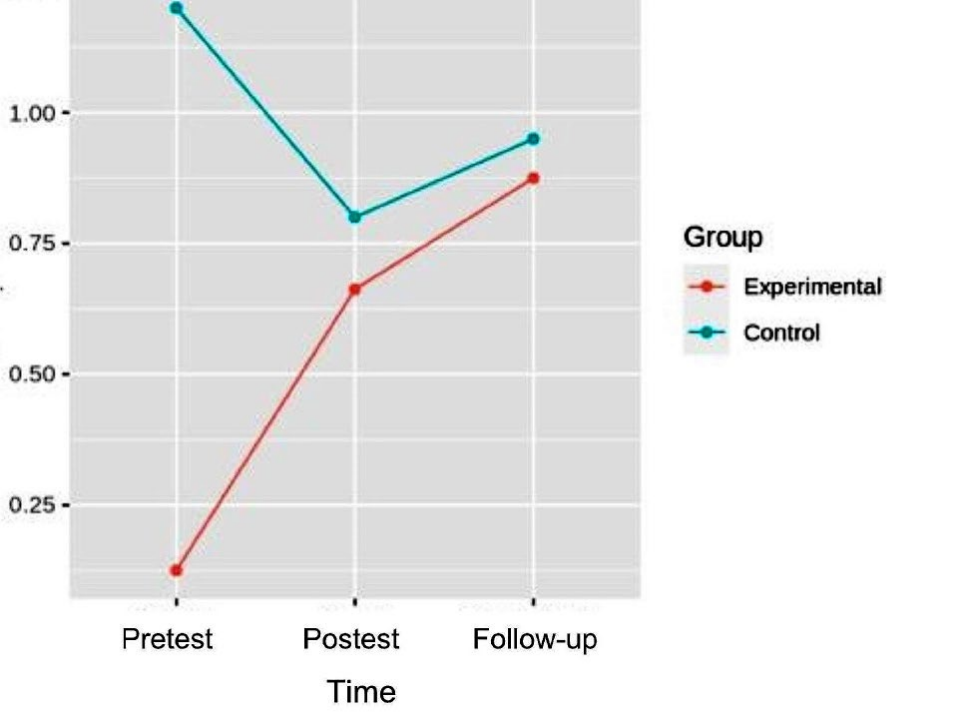
Interaction graph between time and group of right hip extension strength

In the extension strength, in the pretest-posttest and follow-up analysis of variance, differences were found with respect to the interaction [F (2.32) = 4.672, *p* = 0.017, η^2^ = 0.035], in right hip extension strength. (Fig. 5). In the left hip, no differences were found with respect to the interaction [F (2.32) = 3.061, *p* = 0.061, η^2^ = 0.041]. (Table 3).

#### Hip abduction

**Fig. 6:**
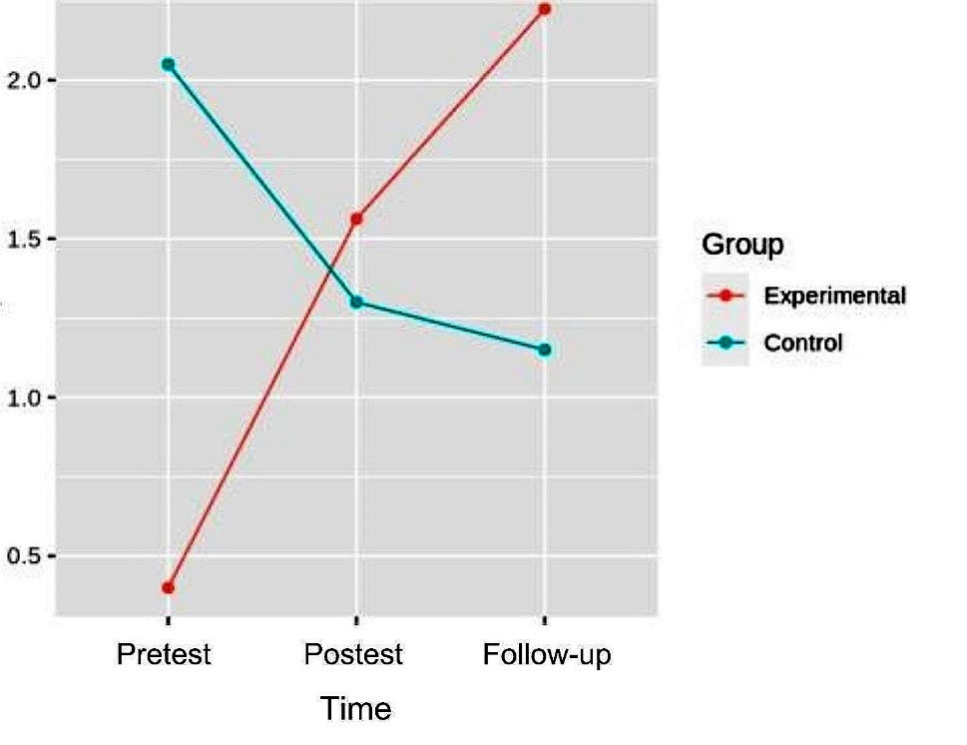
Interaction graph between time and group of left hip abduction strength

**Fig. 7:**
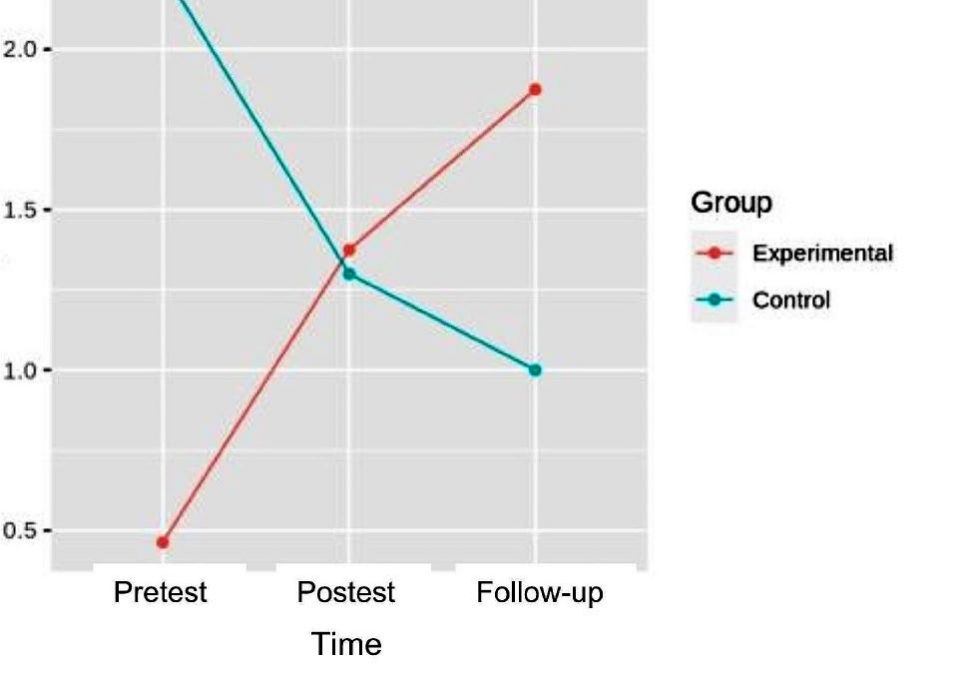
Interaction graph between time and group of right hip abduction strength

In the abduction strength, in the analysis of variance pretest-posttest and follow-up, differences were found with respect to the interaction [*p* = 0.014; η^2^ = 0.179] in the left hip. In the abduction strength of the right hip, differences were also found with respect to the interaction [F (2.32) = 8.721, *p* = 9.481e-04, η^2^ = 0.196]. (Figures 6 and 7). (Table 3).

#### Knee flexion

**Fig. 8:**
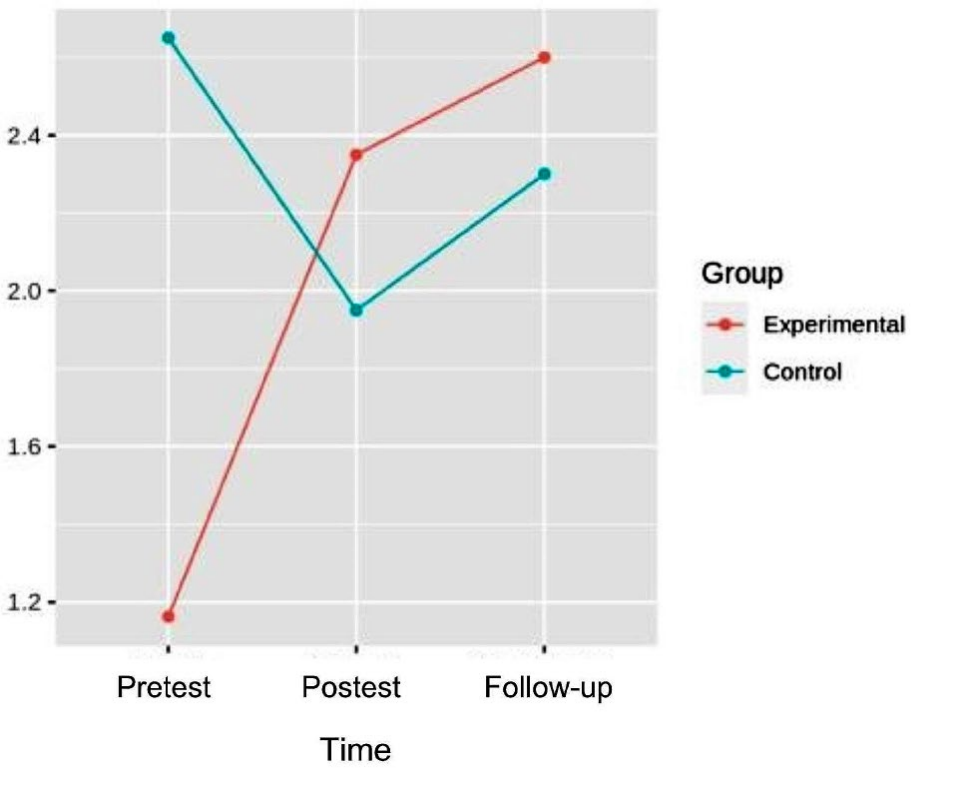
Interaction graph between time and group of left knee flexion strength

**Fig. 9:**
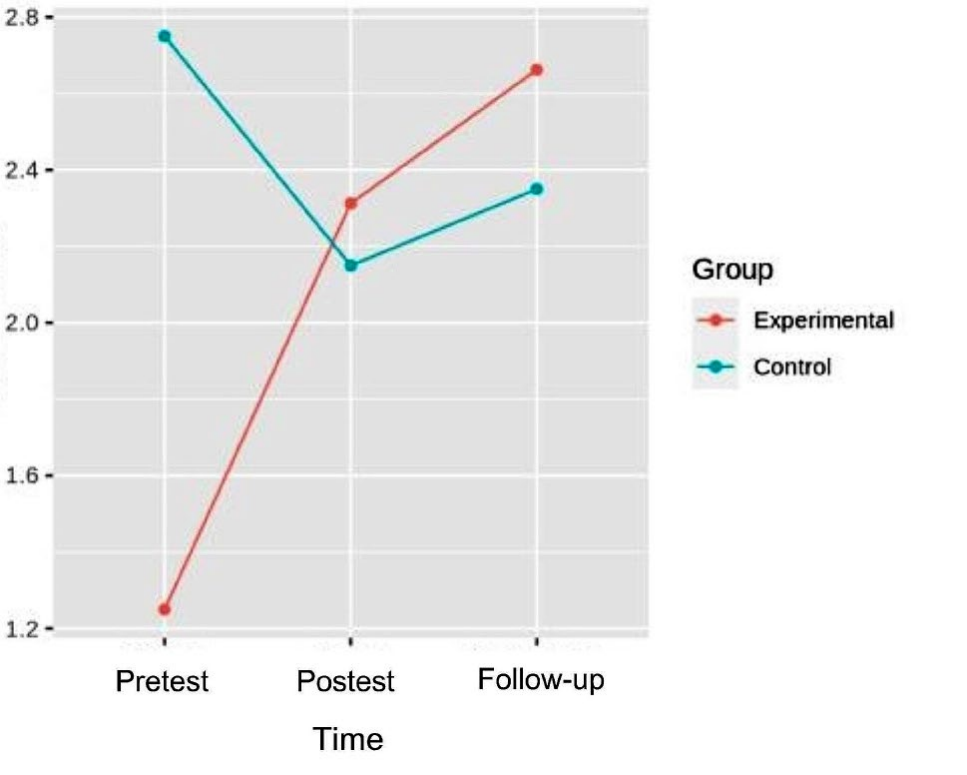
Interaction graph between time and group of right knee flexion strength

In the flexion strength, in the ANOVA differences were found in the strength of the left knee with respect to the group x time interaction [F (2.32) = 9.762, *p* = 4.899e-04, η^2^ = 0.222]. In right knee strength, differences were also found in the group x time interaction [F (2.32) = 4.867, *p* = 1.427e-02, η^2^ = 0.147]. (Figures 8 and 9). (Table 3).

#### Knee extension

In the extension strength, in right knee extension, no differences were found with respect to the interaction [F (2.32) = 0 0.389, *p* = 0.680, η^2^ = 0.005], as in left knee extension (*p* = 0.474). (Table 3)

**Table 3:** Comparisons between groups of hip and knee strength

	Pretest Mean (SD)		Posttest Mean (SD)		Follow-up Mean (SD)			Pretest-post-test and follow-up analysis of variance	
Strength	EG (*n* = 10)	CG (*n* = 10)	EG (*n* = 10)	CG (*n* = 10)	EG (*n* = 8)	CG (*n* = 10)	*p*		η^2^
Left hip flexion	1.46 (0.78)	3.62 (1.97)	2.35 (0.26)	3.10 (1.15)	3.56 (2.29)	3.25 (1.27)		2.350e-03	0.128
Right hip flexion	1.25 (0.75)	3.45 (1.86)	2.45 (0.28)	2.85 (1.18)	3.19 (1.46)	3.15 (1.16)		1.478e- 03	0.142
Left hip extension	0.10 (0.32)	0.95 (1.71)	0.60 (0.94)	0.70 (1.03)	1.12 (1.79)	0.75 (1.23)		0.061	0.041
Right hip extension	0.10 (0.32)	1.20 (1.86)	0.53 (0.82)	0.80 (1.21)	0.88 (1.22)	0.95 (1.32)		0.017	0.035
Left hip abduction	0.32 (0.53)	2.05 (1.88)	1.35 (0.82)	1.30 (0.98)	2.22 (1.77)	1.15 (1.11)		0.014	0.179
Right hip abduction	0.42 (0.50)	2.25 (1.72)	1.25 (0.75)	1.30 (1.01)	1.88 (1.58)	1 (0.97)		9.481e-04	0.196
Left knee flexion	1.18 (0.72)	2.65 (1.25)	2.23 (0.48)	1.95 (0.86)	2.60 (0.92)	2.30 (0.71)		4.899e-04	0.222
Right knee flexion	1.25 (0.68)	2.75 (1.89)	2.20 (0.48)	2.15 (0.63)	2.66 (1.03)	2.35 (0.78)		1.427e-02	0.147
Left knee extension	1 (0.85)	1.84 (1.73)	1.99 (0.62)	2.20 (1.7)	2.09 (1.26)	2.35 (1.73)		0.474	------
Right knee extension	1.10 (0.84)	1.95 (1.59)	2.40 (1.26)	2.45 (2.05)	2.05 (1.7)	2.55 (2.34)		0.680	0.005

SD: standard deviation; EG: Experimental Group; CG: Control Group; n: sample

### ROM

#### Knee flexion

In the flexion movement of the left knee, the pretest-posttest and follow-up analysis of variance showed differences with respect to interaction (*p* = 0.005; η^2^ = 0.071). with medium effect size. In the ROM of right knee flexion, the analysis showed differences with respect to interaction (*p* = 0.005; η^2^ = 0.053), with medium and small effect sizes respectively. (Table 4).

#### Knee extension

In the extension movement in the left knee, the ANOVA pretest-posttest and follow-up showed no differences with respect to interaction [F (2.32) = 0.009, *p* = 0.990]. In the right knee extension ROM, no differences were found with respect to interaction [F (2,32) = 0.477, *p* = 0.624]. (Table 4)

**Table 4:** Comparisons between groups of knee ROM

	Pretest Mean (SD)		Posttest Mean (SD)		Follow-up Mean (SD)		Pretest-post-test and follow-up analysis of variance	
ROM	EG (*n* = 10)	CG (*n* = 10)	EG (*n* = 10)	CG (*n* = 10)	EG (*n* = 8)	CG (*n* = 10)	*p*	η^2^
Left knee flexion	126.50 (11.07)	127 (15.67)	119 (7.38)	128 (11.35)	130 (0)	128 (10.33)	0.005	0.071
Right knee flexion	128 (6.32)	127 (15.67)	121 (3.16)	127 (13.37)	130 (0)	128 (10.33)	0.005	0.053
Left knee extension	-2.30 (3.89)	-2 (6.32)	-1.30 (2.16)	-1.50 (4.74)	-0.62 (1.77)	-1 (3.16)	0.990	6.026e-05
Right knee extension	-2.50 (4.25)	-3 (9.49)	-1.30 (2.16)	-3.50 (9.44)	-0.62 (1.77)	-2.50 (6.35)	0.624	0.002

SD: standard deviation; EG: Experimental Group; CG: Control Group; n: sample; ROM: range of motion

## Discussion

The objective of the present study was to evaluate the comparative effectiveness of RAGT treatment with Walkbot combined with physiotherapy, compared to isolated physiotherapy treatment, in children and adolescents with CP, to improve gait and increase muscle strength and range of motion in the lower extremities.

In this study we have evaluated walking ability as an integral part of motor development. As we included children who were not yet walking and were at level IV of the GMFCS, we were able to assess their functionality, both in standing and walking through the use of the GMFM-88. Previous studies with robot-assisted gait training also used this scale as outcome measures to assess functional activity [17, 19–21, 27].

In our study, positive changes in the EG have been observed in the GMFM-88, both in the post-test and in the follow-up. Dimension D obtained a significant difference. And in dimension E, no significant differences were observed. Regarding previous studies, some of them found improvements in dimension D and E [19–21, 27]; and one study in dimension E [17]. These studies mentioned included children with GMFCS-ER levels between I and III, and none included level IV, unlike our study. Furthermore, in our study all children had bilateral involvement, and others included unilateral involvement along with bilateral [20], or only unilateral involvement [21].

In this study children were in levels II, III and IV of the GMFCS, and some of them had intellectual disabilities. In the control group, the 50% was in the level II in the GMFCS, while in the experimental group the 46.15% was in the level III, and the 15.38% was in the level II. Also, in the experimental group there were more children with cognitive impairment (61.54%), than in the control group (50%).

Our study is the first clinical trial that compares Walkbot together with physiotherapy with physiotherapy treatments in CP in a European population. A study [25] use Walkbot comparing two groups in which both used the RAGT, without a control group. In our study, significant results were observed in the comparison of the Walkbot treatment together with physiotherapy with physiotherapy treatments in standing, muscle strength, and lower limb ROM. Other CTs that use RAGT report improvement in standing [19–21, 27]. However, previous CTs do not measure strength or ROM like our study.

Some previous clinical trials have demonstrated improvements in walking ability in participants with neurological damage, such as stroke and spinal cord injury, who used a walking robot in their rehabilitation [10–14]. In children with CP, reviews and meta-analyses have not found significant differences in the use of the walking robot compared to other physiotherapy treatments aimed at rehabilitating walking function [22, 28, 29). In a recent meta-analysis that included 8 clinical trials (2 non-randomized), no significant differences were found compared to physical therapy treatments [22].

Regarding limitations, the groups were not randomized, since participants who were willing to travel to the place where the treatment was carried out were assigned to the EG, and the final sample size was reduced compared to what was expected. On the other hand, we have evaluated variables related to body structure, function and activity, but not to participation, according to the ICF framework [30].

In future research, it would be advisable to conduct RCTs, with homogeneous groups in terms of levels of impairment in the GMFCS-ER, and with larger samples. It would also be convenient to compare the treatment with specific physiotherapy treatments, and to contrast whether treatment times are shortened by including the use of the walking robot.

## Conclusions

Physiotherapy treatment including the RAGT Walkbot has shown improvements in standing, lower limb muscle strength, and knee ROM in children and adolescents with CP studied, compared to physiotherapy treatments without RAGT. This improvement may predispose to an increase in the activity and participation of this population in their environment and in their ability to carry out their daily activities. On the other hand, no differences were found between both treatments in dimension E of the GMFM in the study participants.

## Data Availability

No datasets were generated or analysed during the current study.

## Abbreviations

CP: Cerebral Palsy
RAGT: Robotic-assisted gait training
GMFM: Gross Motor Function Measure
GMFCS-ER: Gross Motor Function Classification System – Expanded & Revised
EG: Experimental Group
CG: Control Group
ROM: Range of Motion
RCT: Randomise Clinical Trial
